# nf-core/circrna: a portable workflow for the quantification, miRNA target prediction and differential expression analysis of circular RNAs

**DOI:** 10.1186/s12859-022-05125-8

**Published:** 2023-01-24

**Authors:** Barry Digby, Stephen P. Finn, Pilib Ó Broin

**Affiliations:** 1 School of Mathematical and Statistical Sciences, National University of Ireland, Galway, Ireland; 2 Department of Histopathology and Morbid Anatomy, Trinity Translational Medicine Institute, Dublin, Ireland

**Keywords:** Circular RNA, ceRNA, Nextflow, nf-core, Portable analysis workflow

## Abstract

**Background:**

Circular RNAs (circRNAs) are a class of covalenty closed non-coding RNAs that have garnered increased attention from the research community due to their stability, tissue-specific expression and role as transcriptional modulators via sequestration of miRNAs. Currently, multiple quantification tools capable of detecting circRNAs exist, yet none delineate circRNA–miRNA interactions, and only one employs differential expression analysis. Efforts have been made to bridge this gap by way of circRNA workflows, however these workflows are limited by both the types of analyses available and computational skills required to run them.

**Results:**

We present nf-core/circrna, a multi-functional, automated high-throughput pipeline implemented in nextflow that allows users to characterise the role of circRNAs in RNA Sequencing datasets via three analysis modules: (1) circRNA quantification, robust filtering and annotation (2) miRNA target prediction of the mature spliced sequence and (3) differential expression analysis. nf-core/circrna has been developed within the nf-core framework, ensuring robust portability across computing environments via containerisation, parallel deployment on cluster/cloud-based infrastructures, comprehensive documentation and maintenance support.

**Conclusion:**

nf-core/circrna reduces the barrier to entry for researchers by providing an easy-to-use, platform-independent and scalable workflow for circRNA analyses. Source code, documentation and installation instructions are freely available at https://nf-co.re/circrna and https://github.com/nf-core/circrna.

**Supplementary Information:**

The online version contains supplementary material available at 10.1186/s12859-022-05125-8.

## Background

CircRNAs are a class of non-coding RNAs (ncRNAs) formed by the back-splicing of precursor messenger RNA (pre-mRNA) to create a single-stranded covalently closed RNA loop structure. Initially discovered in plant viroids [1], yeast mitochondrial RNAs [2], and the hepatitis $\delta$ virus [3], circRNAs were believed to be a by-product of aberrant splicing or intermediates discarded from intron-lariat debranching [4, 5], going largely unstudied during the formative years of RNA-Seq due to poly-A selection protocols. Recent advancements in bioinformatics methods have facilitated the detection of circRNAs in RNA-Seq datasets, with interest in the field revitalised when Salzman et al. identified circRNAs in cancer and non-cancer cell lines [6] resulting in a shift in the paradigm of RNA-Seq protocols enabling detection of circRNAs (total RNA-Seq, ribosomal RNA (rRNA) depleted RNA-Seq).

Several characteristics of circRNAs make them ideal candidates as clinical biomarkers for cancers and diseases; lack of 5’ and 3’ ends conferring resistance to RNase degradation granting much higher half-lives than their linear counterparts [6, 7], tissue- and developmental-stage specific expression, and abundance in blood, serum, plasma and exosomes [8, 9]. Recent evidence has accumulated demonstrating circRNAs viability as a biomarker, with reports of circRNAs exhibiting differential expression in gastric cancer [10], colorectal cancer [11], bladder cancer [12], cardiovascular disease [13] and Alzheimer’s disease [14] amongst many others. circRNAs have also been shown to harbour functionally active and evolutionarily conserved microRNA response elements (MREs) within their mature spliced sequence [15, 16], suggesting a regulatory role within the competing endogenous RNA (ceRNA) network by titrating the limited pool of endogenous miRNAs available to mRNAs [17]. One such example is circCCDC66, which is hypothesized to play a protective role for the MYC oncogene by binding to miR-33b and miR-99 in colorectal cancer. Knockdown of circCCDC66 displayed reduced MYC expression, whilst administration of miR-33b and miR-99 reversed the knockdown effect, inferring circCCDC66 is capable of modulating mediating MYC expression via the ceRNA network [18].

Regarding the quantification, miRNA target prediction and differential expression analysis of circRNAs, several workflows currently exist (Table 1). However, the majority of these workflows are limited by the type of analysis they can perform, often acting as either: (1) a downstream analysis toolkit requiring the user to supply circRNA quantification results, or (2) a workflow offering multiple quantification tools to generate high confidence circRNA calls. Both scenarios necessitate in-house computational expertise to supplement the missing elements of the analysis, resulting in a high barrier to use for both novice and seasoned researchers alike due to the non-standardised input requirements to each workflow.

**Table 1:** List of current circRNA analysis workflows

Pipeline	Published	Dependencies	CQ	MP	DE	Installation
CirComPara [19]	2017	Python, R or Docker	$\checkmark$	$\times$	linear RNA	Source code, DockerHub
CirComPara2 [20]	2021	Python, R or Docker	$\checkmark$	$\times$	$\times$	Source code, DockerHub
DEBKS [21]	2021	Python	$\times$	$\times$	$\checkmark$	Source code, Conda, pip
FcircSec [22]	2020	R	$\times$	$\times$	$\times$	CRAN
circRNAProfiler [23]	2020	R	$\times$	$\checkmark$	$\checkmark$	Bioconductor
circtools [24]	2019	Python, R	$\checkmark$	$\times$	$\checkmark$	Conda, Pypi
circMeta [25]	2020	R	$\times$	$\times$	$\checkmark$	Source code via devtools
circRNAwrap [26]	2019	Perl, Python, R	$\checkmark$	$\times$	$\checkmark$	Manual Installation
Ularcirc [27]	2019	R, R Shiny	$\times$	$\checkmark$	$\times$	Bioconductor
nf-core/circrna	2023	Nextflow, java,Docker/Apptainer	$\checkmark$	$\checkmark$	$\checkmark$	DockerHub

*CQ* circRNA quantification, *MP* miRNA prediction, *DE* differential expression

To address these shortcomings, we present nf-core/circrna, a workflow for the quantification, miRNA target prediction and differential expression analysis of circRNAs in RNA-Seq data developed using nextflow [28]. The pipeline has been developed under the specifications of the nf-core framework [29], ensuring best practices in pipeline development and maintenance.

## Implementation

### nf-core/circrna architecture

The workflow is modularised via processes, whereby each process represents an individual task within the workflow. Nextflow automatically distributes each process in an isolated environment where task execution is performed within a container, ensuring efficient utilization and scaling of computing resources user-defined via configuration files. By utilising containers, the arduous task of installing and satisfying software dependencies has been completely removed, thereby facilitating rapid ‘out of the box’ deployment and ensuring reproducible analyses through pinned software versions.

nf-core/circrna is configured to run on local machines, high performance compute clusters and cloud infrastructures alike, facilitating most deployment scenarios via customisable configuration profiles. Profiles for popular platforms (AWS, Azure, Google Cloud) and multiple research institutions can be found at (https://github.com/nf-core/configs). In the event ones institution is not represented, users are encouraged to fork the nf-core/circrna repository and make the appropriate changes to the configuration profile, reflecting the task scheduler and min/max computational resources available before attempting to deploy the workflow.

Comprehensive documentation including instructions to perform a test dataset analysis can be found at https://nf-co.re/circrna. Furthermore, the nf-core community harbours an active Slack channel where users can contact the authors directly with queries regarding the workflow, or open an issue on the workflows github page. Three dependencies are required to run nf-core/circrna: Java ($>=8$), the latest version of nextflow [28] and a software container client such as Docker [30] or Apptainer (formerly Singularity) [31].

### nf-core/circrna functionality

**Fig. 1:**
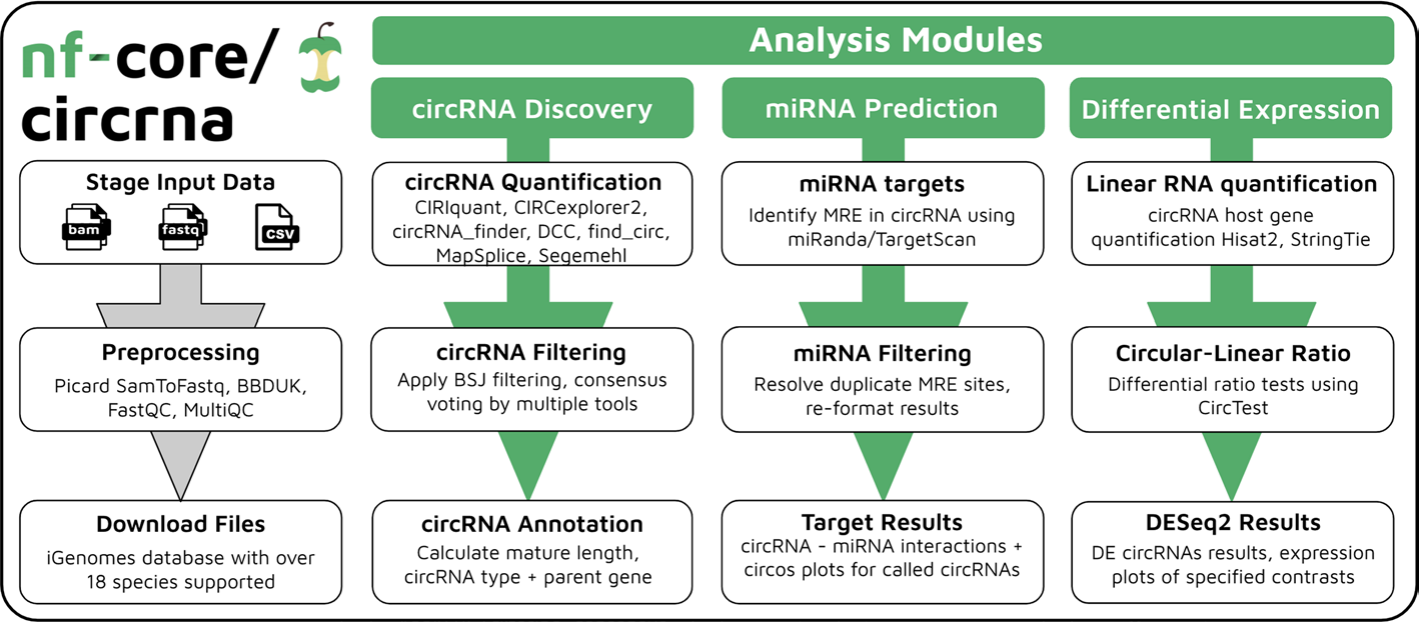
Schematic overview of nf-core/circrna analysis modules

The functionality of nf-core/circrna can be summarized by three core analysis modules: (1) circRNA discovery and quantification (2) miRNA target prediction and (3) differential expression analysis (Fig. 1). The circRNA discovery module is required, however users may choose to include or omit the miRNA prediction or differential expression modules to suit their analysis needs. Pre-processing steps are optional, depending on the format and quality of the input data supplied to the workflow.

The pipeline accepts paired-end RNA-Seq data in the form of FASTQ files or BAM files as inputs to the workflow. In the event BAM files are supplied, the workflow will automatically revert the BAM files to paired end FASTQ files for back-splice junction detection. Summary statistics of read quality, sequencing artefacts and adapter contamination are generated using FastQC [32], with an optional parameter available to perform adapter removal and read filtering using BBDUK [33], allowing the user to pass raw or trimmed reads to the circRNA discovery module.

Reference annotation files (FASTA, GTF) are automatically downloaded via the Illumina iGenomes database, with 18 species currently supported by the workflow in ENSEMBL, UCSC and NCBI annotations where available. In addition to providing correctly formatted reference files to the workflow, the iGenomes database hosts genome index files compatible with multiple aligners, significantly reducing the computational cost of analyses. In the event the user is working with a non-model organism, reference files can be supplied to the workflow manually.

### circRNA discovery

nf-core/circrna currently offers seven circRNA quantification tools: CircExplorer2 [34], circRNA_finder [35], DCC [36], CIRIquant [37], MapSplice [38], find_circ [7] and Segemehl [39]. As both CIRCexplorer2 and CIRIquant can parse the outputs from multiple aligners, we define CIRCexplorer2 as utilising STAR [40] 2-pass mode prior to parsing, whilst CIRIquant utlises its canonical BWA [41]–CIRI2 [42] workflow. The user may select one, all or any combination of circRNA tools available in the workflow by providing a list to the ‘--tool’ parameter. In the scenario where more than one quantification tool has been selected, the parameter ‘--tool_filter’ accepts as input a positive integer *n* to perform consensus based filtering on called circRNAs, requiring circRNAs to be called by at least *n* quantification tools during the count matrix generation step. Users can also apply one of two aggregation functions to circRNAs called by multiple tools using the ‘--duplicates_fun’ parameter, which accepts ‘max’ or ‘mean’ returning the highest count value or averages, respectively. To reduce spurious circRNA candidates, the parameter ‘--bsj_filter’ filters circRNAs based on the number of reads spanning the back-splice junction site during parsing of the raw quantification tool outputs.

Candidates that satisfy the filtering thresholds are passed to an in-house reference-guided circRNA annotation script detailing circRNA type, parent gene, underlying exons and mature spliced length, producing an extended BED-12 output file. The in-house script considers the four following cases for circular candidates: *Circular candidates start and end positions overlap exon boundaries*: If circular candidates start and end positions perfectly overlap exon boundaries, the candidate is marked as an exonic circRNA, or simply ‘circRNA’. *Circular candidates start and end positions imperfectly overlap exon boundaries*: If the start and end positions are suitably within range (< 200bp) of an exon boundary, the candidate is assumed to be a circRNA. However, if one of the start or end position reside outside of the range of an exon boundary (> 200bp), the circular candidate is assumed to be an exon-intron circRNA (EI-circRNA). Users can toggle the 200bp distance via the ‘--exon_boundary’ parameter. *Circular candidate resides within a gene, but does not overlap any exon boundaries*: These circular candidates are marked as intronic circular RNAs (ciRNAs). *Circular candidates do not overlap any features in the reference GTF file*: Such candidates are assumed to be intergenic circRNAs and will have an ‘NA’ description for underlying transcripts and parent gene in the output file. Due to the scripts reliance on reference annotations, the workflow is suitable only for analyses of species for which a reference GTF file exists.

Finally, utilising the information held in the BED12 file, the circRNA_discovery module outputs the mature spliced sequence of each called circRNA in FASTA format and generates a circRNA count matrix for downstream statistical testing, which are used as inputs for the ‘miRNA_prediction’ and ‘differential_expression’ modules, respectively. To access the raw output files generated by each quantification tool, users can toggle the ‘--save_intermediates boolean parameter to copy files from the work directory to the specified results directory.

### miRNA prediction

To elucidate the regulatory role of circRNAs, nf-core/circrna offers a miRNA prediction module to identify miRNA response element (MRE) sites within the mature spliced sequence and back-splice junction site of detected circRNAs using both miRanda [43] and TargetScan [44] prediction algorithms. To reduce the number of spurious calls, target miRNAs must be called by both target prediction algorithms. The predictions are output in a results file per sample, detailing circRNA-miRNA node/edge membership complete with miRanda scores, minimum free energy (Kcal/mol) and site type (6mer, 7mer-m8, 7mer-A1, 8mer). Should the user wish to apply strict post-hoc filtering, we suggest users remove 6mers and miRNAs with a minimum free energy score of $>=-20.00$ Kcal/Mol [45].

### Differential expression analysis

nf-core/circrna offers a differential expression module to detect differentially expressed circRNAs and model changes in circRNA expression relative to its host gene guided by the phenotype.csv file provided by the user. It is important to note that pre-filtered datasets with linear RNAs removed are not suitable for the differential expression module.

Firstly, linear RNAs are quantified using Hisat2 [46] and StringTie [47] to determine both host gene expression and library size factors, which are utilised to normalize the circRNA count matrix. Following normalization, quality control plots are generated to assess sample heterogeneity: analysis of principal components via PCA plots and hierarchical clustering of samples via heatmaps and dendograms. The workflow automates differential expression analysis for all possible combinations of the levels provided under the column ‘condition’, whilst controlling for additional covariates added to the phenotype design file using DESeq2 [48]. For each contrast, the workflow reports both up-regulated and down-regulated circRNAs, plots of global expression patterns via heatmaps, volcano and MA plots, and the distribution of adjusted and non-adjusted p-values. Furthermore, for each differentially expressed circRNA returned by the contrast, boxplots are generated displaying the normalized expression between experimental conditions. To compliment the circRNA results, the workflow concurrently performs linear RNA-Seq differential expression analysis.

The CircTest [24] R package employs a beta-binomial model to test differences between circRNA and host gene expression to identify cases of enrichment or depletion of circRNA relative to the linear transcripts from the same host genes. The module outputs PDF plots of circRNA-host gene line plots, ratio plots and a table of the statistical results for users.

## Results and discussion

### nf-core/circrna demonstration full dataset

To showcase the utility of nf-core/circrna in a single execution, we recapitulated a previously published study investigating the effect of mutant RNA binding homolog FUST-1 on circRNA biogenesis in total RNA seq samples of *C. elegans* (PRJNA742881) [49] Table 2.

**Table 2:** Run information using PRJNA742881 dataset

Project accession	Genome build	Run time	CPU hours	Sample ID	Number of reads
PRJNA742881	WBcel235	18 h 21 m 20 s	1566	N2_1	72651039
				N2_2	92546415
				N2_3	82870805
				fust1_1	70194536
				fust1_2	89945035
				fust1_3	86470345

Setting up the analysis required minimal effort—raw sequencing data was retrieved using nf-core/fetchngs [50] by providing the sequencing archive project accession number, a sample CSV file was constructed specifying the sample ID’s and sample paths, a phenotype CSV file was designed detailing sample metadata for DESeq2 and both the ‘WBcel235’ and ‘cel’ strings were passed to the workflow to automatically download ENSEMBL reference annotation and miRNA database files, respectively. The analysis was run on a local SLURM HPC cluster with maximum ncpu and cpu memory limits set to 8 and 60 GB, representing a moderate level of resources feasible for most research labs.

**Fig. 2:**
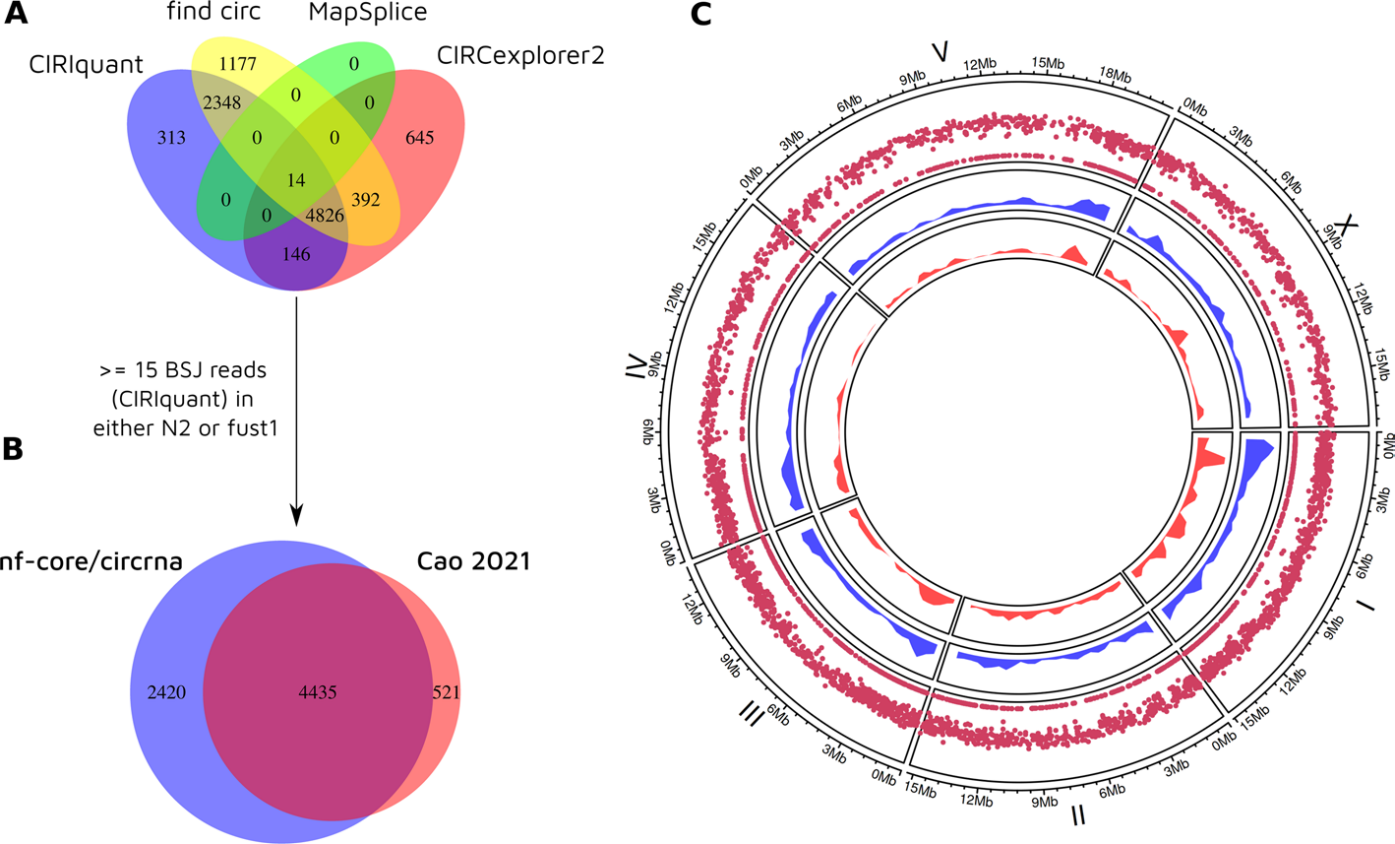
Comparison of filtered circRNAs **A** circRNAs called by each quantification tool in this study. **B** common circRNAs in this study versus PRJNA742881. Filtered circRNAs were required to have at least 15 BSJ reads called by CIRIquant. **C** Genomic distribution of 4435 common filtered circRNAs (outer layer), 2420 additional circRNAs discovered by nf-core/circrna (middle layer) and 521 circRNAs unique to PRJNA742881 (inner layer)

**Fig. 3:**
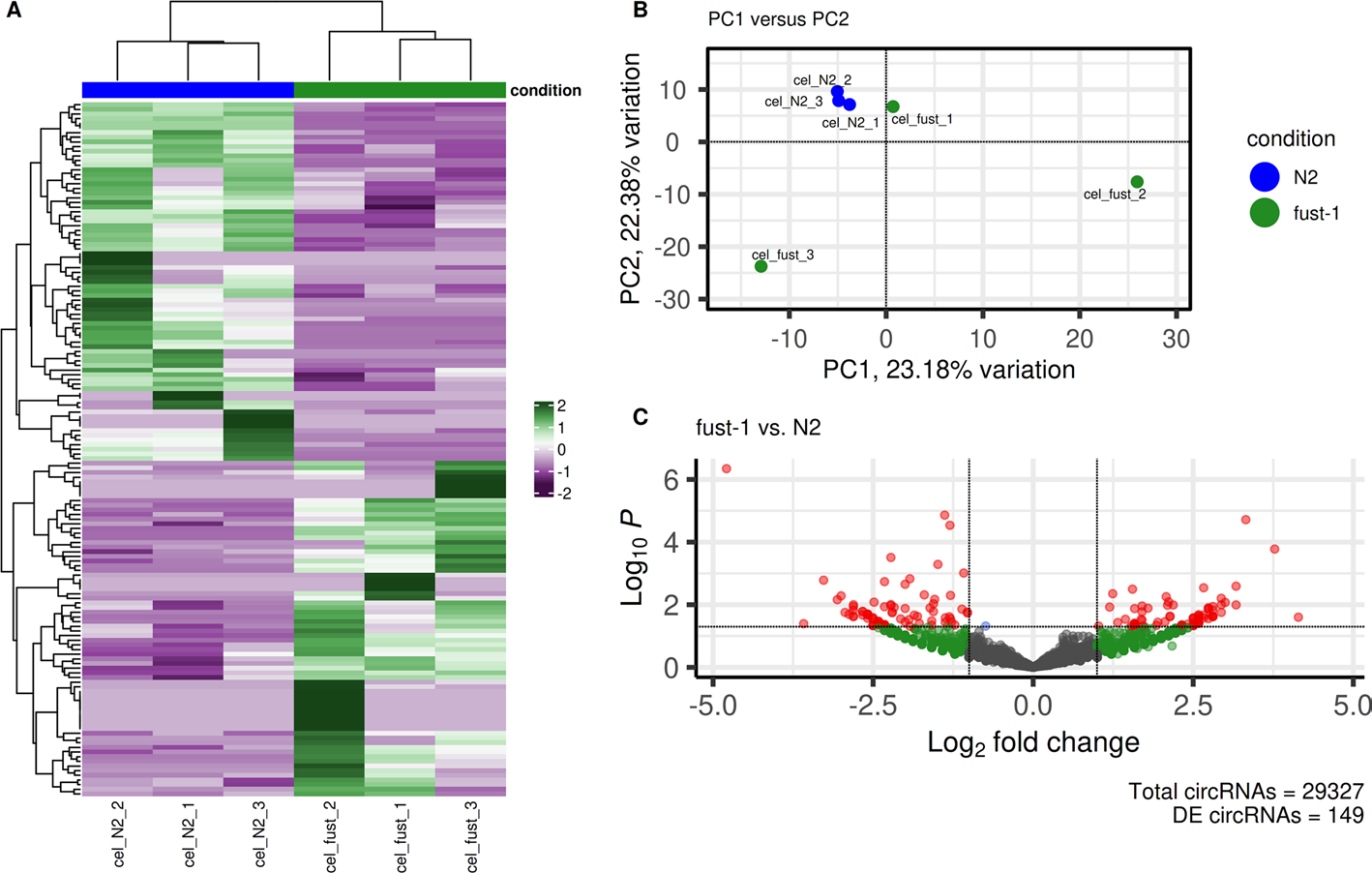
**A** Heatmap of differentially expressed circRNAs. **B** Principal component bi-plot of PRJNA742881. **C** Volcano plot of differentially expressed circRNAs

Based on the results of the simulated dataset (Table 3), CIRCexplorer2, CIRIquant, find_circ and MapSplice were chosen for circRNA quantification due to their high F1 scores (> 91%). The original analysis by Cao et. al used CIRCexplorer2, DCC and CIRI2. The justification for not using CIRI2 and DCC are as follows: CIRIquant is the successor to CIRI2, acting as a wrapper script around CIRI2 and as such, CIRI2 remains the underlying circRNA detection software. DCC requires an extremely high amount of RAM (analysis failed with 120GB RAM allocated to DCC processes), most likely caused by the exceptionally high coverage of circRNAs in the RNase R Treatment, Polyadenylation, and Poly(A)$+$ RNA Depleted (RPAD) dataset. Due to it’s computational inefficiency and high false positive rate in Table 3, we reasoned that the analysis would not suffer due to its omission.

In less than 19 h, a single execution of nf-core/circrna produced a comprehensive profile of circRNAs in N2 and fust1 *C. elegans* samples, recovering 4435 filtered circRNAs detected by Cao et al. and an additional 2420 circRNAs that passed stringent filtering parameters (Fig. 2). A selection of outputs from the ‘differential_expression’ module are given in Fig. 3, demonstrating the workflow’s ability to generate interpretable results for end users.

### nf-core/circrna performance evaluation

#### Computational cost

**Fig. 4:**
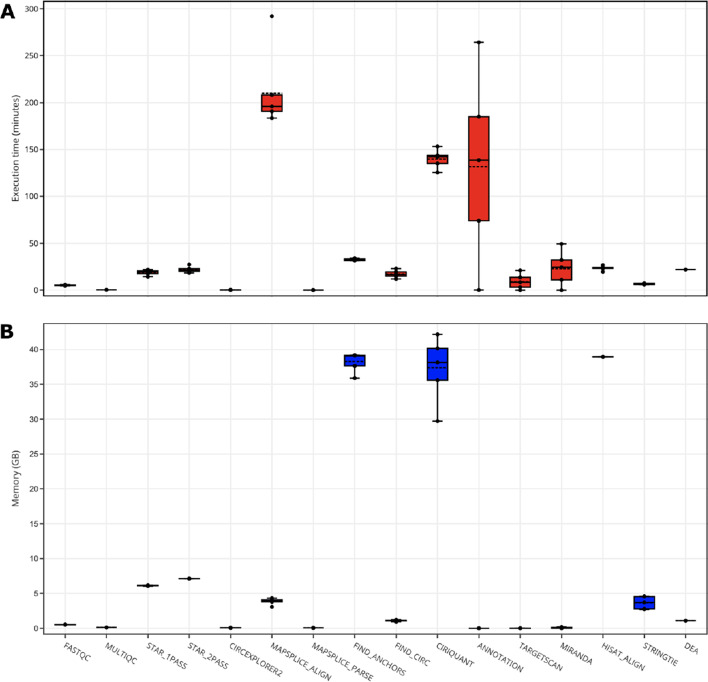
nf-core/circrna performance results (PRJNA742881). **A** Time elapsed per-process in minutes. **B** RAM memory consumed per-process in gigabytes

To assess the performance and resource consumption of nf-core/circrna, the run-time and memory (RAM) usage during the analysis of PRJNA742881 was recorded for each process (Fig. 4) with trivial tasks such as file reformatting are omitted from the figure. Per-process usage statistics revealed the most memory intensive processes were the alignment steps for both find_circ and CIRIquant (38.26 GB, 37.37 GB). All other processes in the workflow required minimal resources (< 10 GB). With respect to process run-times, the alignment steps for MapSplice and CIRIquant averaged 209 and 139 min, respectively. The customised annotation script averaged a 131 min run time, however this process is sensitive to the number of circRNA candidates provided to the process, which can be refined using the ‘--bsj_reads’ parameter. Overall, the performance showcases the efficiency of nf-core/circrna in appropriating tasks in a timely and memory efficient manner, thus making the pipeline deployable on most computing environments.

#### Quantification tool performance

To assess the performance of nf-core/circrna and it’s constituent quantification tools, the workflow was run on a simulated dataset to calculate precision, sensitivity and F1 score performance metrics. The simulated circRNA dataset comprising 1,450,328 synthetic paired-end reads of 101 bp in length and 350 bp insert size was derived using an overhauled version of CIRIsimulator.pl [42] designed by Zeng et al. [51]. This re-worked version of the tool can accept as input circRNA database files from CircBase [52], allowing users to recapitulate *bona fide* circRNAs based on verified back-splice junction coordinates as opposed to the generation of synthetic circRNA reads via the joining of 2 non-collinear exons at random.

7445 glioblastoma circRNAs were downloaded from circRNADb [53] and reformatted to conform to CircBase specifications before being supplied as inputs to CIRIsimulator.pl. A total of 7267 glioblastoma circRNAs were generated by the tool for use in the analysis. As there are no true negatives in the simulated dataset, and all non-true positives are viewed as a false positive, the fraction of relevant instances retrieved by a tool is given by precision, the fraction of relevant instances retrieved relative to the number available instances is given by sensitivity, and the F1 score represents the harmonic mean of precision and sensitivity. In the event a circRNA was called on both strands, we took the decision to.

The results of the simulated analysis are given in Table 3. It is important to note sensible filtering parameters were applied to each set of quantification tool results—called circRNAs were required to have at least 2 reads spanning the back-splice junction site. In addition to assessing the performance of each individual quantification tool, circRNAs that had been called by at least two quantification tools via the ‘--tool_filter’ parameter were extracted from each set of individual results. The resulting set produced scores of 99.83%, 96.12% and 97.94% for precision, sensitivity and F1, respectively, demonstrating the efficacy of a consensus based approach to circRNA quantification.

**Table 3:** Performance metrics of nf-core/circrna quantification tools on the simulated glioblastoma dataset

Tool	Detected	True positives	Precision (%)	Sensitivity (%)	F1 (%)	Run time (m)	Memory (GB)
CIRCexplorer2	6270	6259	99.82	86.13	92.47	7	20
CIRIquant	6668	6663	99.93	91.69	95.63	13	65
circRNA_finder	6171	5997	97.18	82.52	89.25	7	20
DCC	8397	6344	75.55	87.30	81.00	34	21
find_circ	6217	6202	99.76	85.34	91.99	6	3
MapSplice	6155	6153	99.97	84.67	91.69	9	6
Segemehl	4655	4558	97.92	62.72	76.46	6	50

**Fig. 5:**
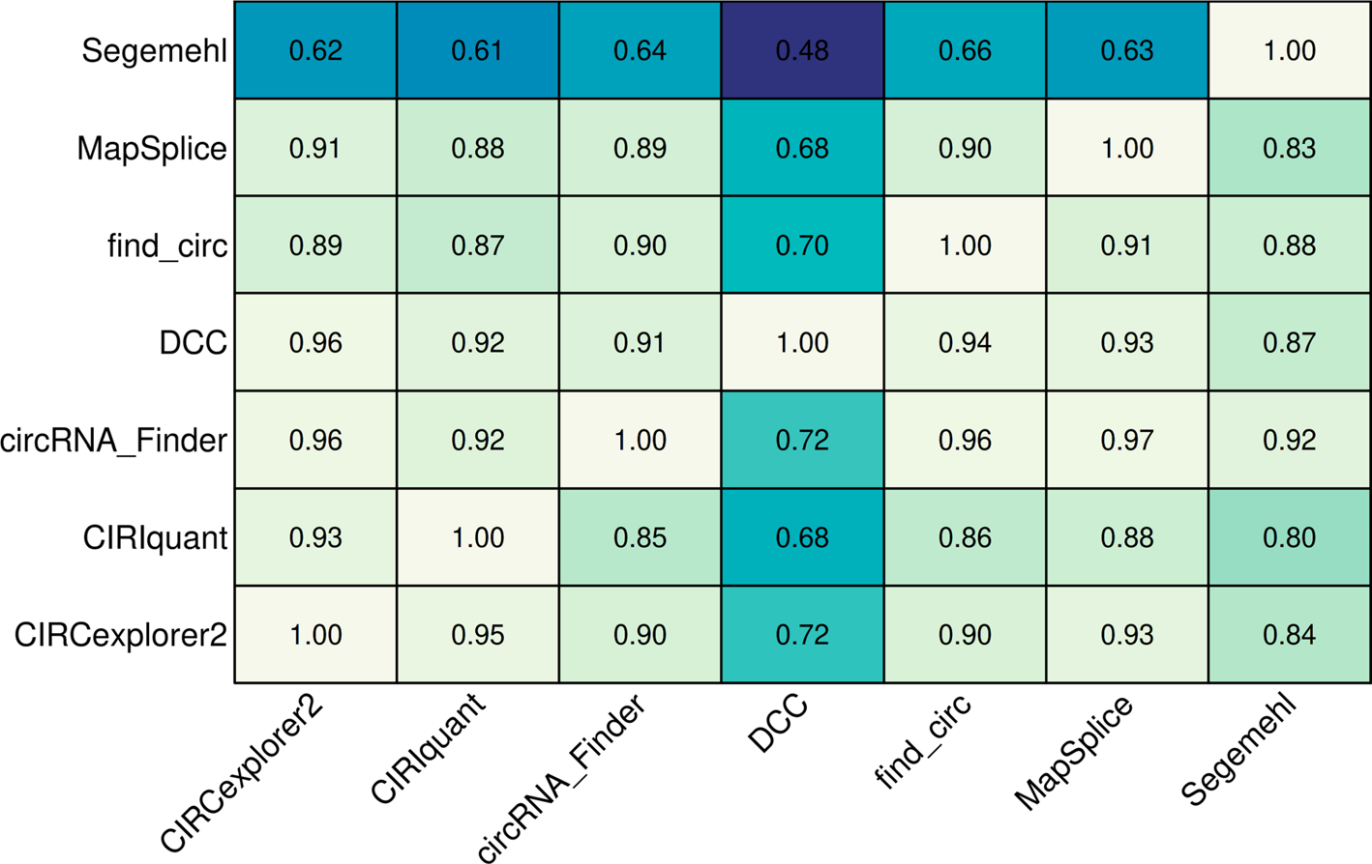
The proportion of common circRNA candidates called by each quantification tool in the simulated glioblastoma dataset

Another useful metric for users to consider when selecting quantification tools for nf-core/circrna is the proportion of common candidates called by each quantification tool. Previously described by Zeng et al. [51], the proportion of common circRNAs shared between two quantification tools *i* and *j* can be represented as *C*(*i*, *j*), with the total number of candidates detected by each tool is $N_i$ and $N_j$, respectively. Thus, for tool *i*, the proportion of common candidates is $P(i,j) = C(i,j)/N_i$ and for tool *j* the proportion of common candidates is $P(j,i) = C(j,i)/N_j$. Pairwise comparisons for all tools in the simulated dataset were generated and represented using a heatmap (Fig. 5). The heatmap can be read in two directions, permitting the bidirectional query of overlapping sets between two quantification tools. When focusing on a specific column, each cell reflects the proportion of common candidates detected by the column tool that were also detected by the corresponding row tool. Conversely, for each row, the cells represent the proportion of common candidates detected by the corresponding column tool that were also recovered by the row tool.

Segemehl detected 4655 circRNAs with 97.92% precision in the filtered simulated dataset however, it was unable to recover a high proportion of circRNAs detected by other tools, indicating a conservative approach to calling circRNAs. In contrast, DCC detected 8397 circRNAs, the highest amount detected by any tool in the simulated dataset. DCC produced a high proportion of circRNAs that were not covered by other tools, suggesting a high rate of false positives—confirmed by its 75.55% precision score. When using the proportion of common candidates to inform quantification tool choices, users should take caution when using DCC by applying sensible filtering parameters to reduce the number of false positives, and supplement Segemehls conservative results with other quantification tools. Encouragingly, CIRCexplorer2, CIRIquant, find_circ and MapSplice results were all found to have high agreement—a high proportion of circRNA candidates detected by these methods were frequently detected by one another—and high precision scores, indicating robust methods for detecting circRNAs.

#### Optimal parameter selection

**Fig. 6:**
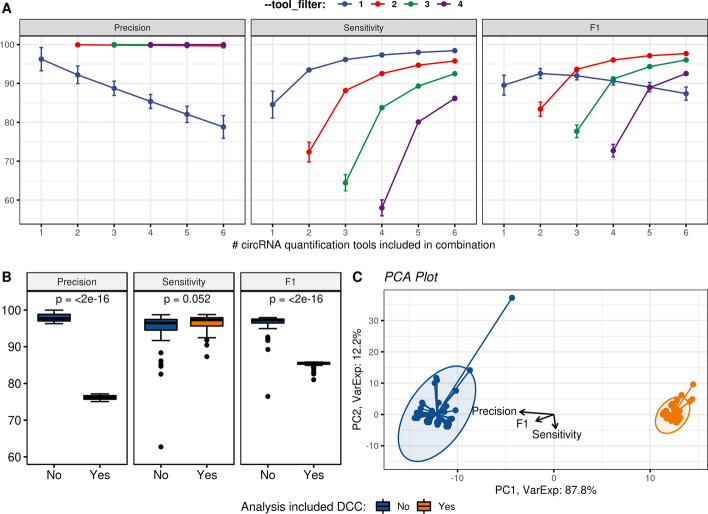
**A** Performance metrics for each combinatorics set of results demonstrating the effect of ‘--tool_filter’ and number of quantification tools included. **B** T-test of all sets including DCC vs. those without DCC for precision, sensitivity and F1 score. **C** Principal component analysis of the sets, displaying the loadings for precision, sensitivity and F1 score

To further advise users on optimal parameter selections for the circrna_discovery module, we performed an analysis on every possible combination of tools using the simulated dataset coupled with iterative filtering using the ‘--tool_filter’ parameter. Our goal was to identify parameters which achieve high performance at the lowest computational cost (i.e requiring the fewest amount of quantification tools in a run). Briefly, a combinatorics approach was taken whereby each set of quantification tool results were used as inputs to generate all possible combinations: $_{n}C_{k} = \frac{n!}{(n-k)!}$ where $n = 6$ and $k = 1 \dots 6$, resulting in 126 unique sets after the removal of duplicates. In Fig. 6A, the x-axis depicts the number of quantification tools included in the combinatoric sets, whilst the y-axis displays the corresponding mean value for precision, sensitivty and F1 score. Each colored line represents the value selected for the parameter ‘--tool_filter’, requiring circRNAs in sets to be called by at least *n* tools.

We observed an inflection point in the lineplot for F1 scores when three quantification tools were selected and ‘--tool_filter’ was set to two. Increasing the number of quantification tools resulted in minimal performance gains, whilst applying stricter filtering thresholds via ‘--tool_filter’ greatly reduced sensitivity. The top ten performing combinations of set size three and ‘--tool_filter 2’ ranked by F1 score are given in Table 4. Full performance metric results for all iterations of ‘--tool_filter’ are available in Additional file 1.

**Table 4:** Top 10 performing quantification tool combinations at the lowest computational cost in the simulated dataset, ranked by F1 score

Combination	Precision (%)	Sensitivity (%)	F1 (%)
CIRCexplorer2,CIRIquant,Segemehl	98.47	97.21	97.83
CIRIquant,MapSplice,Segemehl	98.55	96.99	97.76
CIRCexplorer2,CIRIquant,find_circ	99.59	95.89	97.70
CIRCexplorer2,CIRIquant,MapSplice	99.74	95.54	97.60
CIRIquant,find_circ,MapSplice	99.70	95.57	97.59
CIRCexplorer2,find_circ,Segemehl	98.33	96.57	97.45
CIRCexplorer2,MapSplice,Segemehl	98.50	96.41	97.44
CIRIquant,find_circ,Segemehl	98.37	96.46	97.41
CIRCexplorer2,find_circ,MapSplice	99.61	95.28	97.40
CIRCexplorer2,CIRIquant,circRNA_finder	97.51	97.18	97.35

Furthermore, we were interested in identifying quantification tools that negatively impacted the performance in each of the sets. Clustering analysis of the matrix containing precision, sensitivity and F1 score for all 126 unique sets revealed sets containing results from the tool DCC negatively impacted performance metrics. This was formally tested using a t-test (Fig. 6B) and depicted using principal component analysis whereby the first principal component clearly displays sets containing DCC and negatively correlated with the loadings for precision and F1 score, respectively. DCC is the only quantification tool that utilises STAR 2-pass mode, greatly increasing the sensitivity around splice junction sites at the cost of false positives [54]. Users should take this information into consideration when selecting quantification tools for their own analysis and apply sensible filtering parameters to reduce spurious calls.

## Conclusion

nf-core/circrna is the first portable workflow capable of performing the quantification, miRNA target prediction and differential expression analysis of circRNAs in a single execution. Its ease of use greatly reduces the barrier to entry for users seeking to characterise the role of circRNAs in the ceRNA network. We look forward to the workflows release and subsequent engagement with the research community—as the field of circRNA analyses advances, we will effort to incorporate user feedback, suggestions and requests to maintain the vitality and contemporary status of the workflow.

## Availability and requirements

- Project name:nf-core/circrna
- Project home page: https://nf-co.re/circrna
- Project github page: https://github.com/nf-core/circrna
- Operating system(s): Platform independent
- Programming language: Nextflow DSL2
- Other requirements: Java ($>=8$), nextflow v22.10.4, Docker/Apptainer
- License: MIT License
- Restrictions for academic use: none

## Supplementary Information

**Additional file 1**: Data used to produce Figure 6 provided in four xlsx sheets.

## Data Availability

The workflow is freely available at https://github.com/nf-core/circrna. The full size dataset used in the study is available at PRJNA742881 and code to reproduce the simulated analysis is available at https://github.com/BarryDigby/circRNA_simu.

## Abbreviations

circRNA: Circular RNA
ncRNA: Non-coding RNA
ceRNA: Competing endogenous RNA
rRNA: Ribosomal RNA
CQ: circRNA quantification
MP: miRNA prediction
DE: Differential expression
miRNA: microRNA
MRE: microRNA response element
ciRNA: Intronic circRNA
EI-circRNA: Exon-intron circRNA
RPAD: RNase R Treatment, Polyadenylation, and Poly(A)$+$ RNA Depleted
